# Qualitative and Quantitative Inter-Observer Agreement of Multiparametric Whole-Body MRI in Staging and Follow-Up of Myeloma Patients

**DOI:** 10.3390/diagnostics15212715

**Published:** 2025-10-27

**Authors:** Alice Rossi, Arrigo Cattabriga, Andrea Prochowski Iamurri, Eleonora Antognoni, Irene Azzali, Giacomo Feliciani, Claudio Cerchione, Ilaria Bronico, Danila Diano, Cristina Mosconi

**Affiliations:** 1 Radiology Unit, IRCCS Istituto Romagnolo per lo Studio dei Tumori (IRST) “Dino Amadori”, 47014 Meldola, Italy; 2 Department of Radiology, IRCCS Azienda Ospedaliero-Universitaria di Bologna, 40138 Bologna, Italy; 3 Department of Medical and Surgical Sciences (DIMEC), University of Bologna, 40138 Bologna, Italy; 4 School of Specialization in Radiology, University of Ferrara, 44121 Ferrara, Italy; 5 Unit of Biostatistics and Clinical Trials, IRCCS Istituto Romagnolo per lo Studio dei Tumori (IRST) “Dino Amadori”, 47014 Meldola, Italy; 6 Medical Physics Unit, IRCCS Istituto Romagnolo per lo Studio dei Tumori (IRST) “Dino Amadori”, 47014 Meldola, Italy; 7 Hematology Unit, IRCCS Istituto Romagnolo per lo Studio dei Tumori (IRST) “Dino Amadori”, 47014 Meldola, Italy; claudio.cerchione@irst.emr.it

**Keywords:** multiple myeloma, MY RADS, magnetic resonance imaging, WB-MRI, diffusion weighted imaging, bone disease, inter-observer agreement

## Abstract

**Background**: Whole-body magnetic resonance imaging (WB-MRI) is incorporated into international guidelines and recommendations for imaging patients with multiple myeloma. The aim of this study was to investigate inter-observer agreement of radiologists with different levels of expertise in reporting whole-body MRI performed along MY-RADS criteria in myeloma at baseline and in evaluating response to therapy to better certify the use of these criteria. **Methods**: A total of 52 patients with symptomatic myeloma at first presentation (47) or relapse (5) and planned for a new line of therapy were included. All patients completed baseline whole-body MRI within 1 month prior to starting treatment. A total of 25 patients were evaluated with WB-MRI within 1 month after therapy. Each scan was reported independently by three radiologists using MY-RADS. Differences in observer scores were compared using analysis of variance (ANOVA), and inter-observer agreement was assessed using the intra-class correlation coefficient (ICC). **Results**: Interobserver agreement was excellent for all anatomic regions (> 0.81), both at baseline and at follow-up. Quantitative MRI analysis demonstrated that there was no significant difference in mean observer scores for the whole skeleton, and ICC demonstrated excellent inter-observer agreement at 0.9197 for ROI dimension, 0.94 for ADC values, and 0.98 for rFF%. **Conclusion**: MY RADS has excellent inter-observer agreement in reporting symptomatic myeloma at baseline and follow-up after therapy. In our study, there was no discrepancy between skeletal regions, highlighting specific areas of difficulty.

## 1. Introduction

Multiple myeloma (MM) is the second most common hematologic cancer. It originates from plasma cells, a vital component of the immune system responsible for producing antibodies, which become malignant, proliferating uncontrollably, and leading to various health complications, including bone lesions, anemia, increased infections, and kidney damage or failure [1].

Even if the diagnosis and response assessment primarily depend on the clinical and laboratory criteria [2], imaging plays a crucial role in the management of MM.

Total-body imaging techniques are indispensable for staging and post-treatment re-evaluation, as they allow accurate assessment of bone marrow (BM) involvement. This complication affects 80% to 90% of all MM patients during the course of the disease, impairing their quality of life and representing a major cause of morbidity and mortality [3].

Whole-Body Low-Dose Computed Tomography (WB-LD-CT) is the first option for baseline imaging in MM according to the IMWG guidelines. 18F-fluorodeoxyglucose positron emission tomography-computed tomography (PET-CT) can be considered as an alternative to CT if available and is considered the best imaging tool for response assessment. Whole-body magnetic resonance imaging (WB-MRI) should be used if the CT or PET-CT scans are negative or inconclusive [4].

Comparative studies suggest that WB-MRI is more sensitive than PET-CT for detecting BM infiltration at the MM diagnosis, thanks to high spatial resolution, sensitivity to diffuse pattern, and lack of FDG uptake in 10–15% of MM patients. WB-MRI has proven to have a better sensitivity than PET-CT in identifying myeloma (MM) infiltration, but it demonstrated a poorer specificity [5,6,7]. Some authors believe that the absence of defined procedures and interpretation standards for WB-MRI across centers could be the cause of that, particularly when diffusion-weighted imaging is not used and it is not consistently feasible to differentiate between active and inactive areas, which can result in false-positive results [8]. However, some authors argue that DWI does not solve this problem in distinguishing persistent nonviable lesions from viable lesions [9], but PET-CT, due to the nature of the tracer, is thought to better assess this issue. However, there is still little data in the literature for a real comparison, partly because the inclusion of WB-MRI according to MY-RADS in IMWGs is relatively recent.

The combination of PET-CT and MRI is known to increase lesion detection, with PET-MRI surpassing each modality in sensitivity and diagnostic confidence [10,11]. Furthermore, it has been demonstrated that 11C-methionine PET-CT has higher sensitivity and a stronger correlation with tumor load than PET-CT, detecting more focal lesions and demonstrating greater inter-observer agreement [12].

However, PET-MRI and 11C-methionine PET-CT share similar downsides, which are high costs and limited availability, owing in part to the 11C-methionine’s short half-life, which necessitates on-site cyclotron equipment. In contrast, WB-MRI is more widely available, less expensive, and does not require radiation exposure, making it a more accessible and safe choice for patients.

The Myeloma Response Assessment and Diagnosis System (MY-RADS) Guidelines aim to standardize the acquisition, interpretation, and reporting of WB-MRI in patients with multiple myeloma [13]. These recommendations attempt to increase standardization in imaging methods, allowing for more accurate assessments of disease and treatment response. It allows the acquisition and a means of interpretation of WB-MRI in a multiparametric way, combining the high sensitivity of Diffusion Weighted Images (DWIs) with quantitative information derived from Apparent Diffusion Coefficient (ADC) map and Dixon T1 sequences with relative Fat Fraction (rFF%) map to enhance specificity in the report [14,15].

Moreover, for post-treatment follow-up, the MY-RADS guidelines introduced the Response Assessment Categories (RAC), a five-point Likert scale based on qualitative and quantitative image assessment. DWI and ADC are already used clinically as qualitative indicators of disease presence, progression, or response assessment, and recent publications highlight how relative fat fraction (rFF%) evaluation can further aid in the differential diagnosis of malignant focal lesions [16,17,18,19].

In this scenario, the data published so far on whole-body quantitative imaging are particularly promising, especially for evaluating response to therapy. Early changes in rFF% have been shown to predict response, and it seems likely that ADC and rFF% will be complementary metrics [20,21].

At the time of writing, there is a limited number of studies comparing the repeatability of MY-RADS in bone disease, and they focus solely on baseline WB-MRI [22].

Currently, there are no studies comparing the reproducibility of MY-RADS for response assessment, and there is a limited number of studies evaluating the repeatability of the quantitative evaluation of ADC and rFF% in myeloma [23,24].

This study aims to evaluate the reproducibility of MY-RADS assessments among radiologists with different expertise in WB-MRI. Additionally, by evaluating the inter-reader agreement of ADC and rFF% values among radiologists, we seek to further investigate the methodology’s reproducibility and reliability, which is crucial for early disease detection, patient stratification-risk, and evaluation response to therapy, ultimately leading to improved survival outcomes.

## 2. Materials and Methods

### 2.1. Study Population

All patients who have been referred to IRST from September 2020 to May 2023 for a WB-MRI and enrolled into the Accu-mRI trial (L1P788_IRST) have been retrieved. Only those who met the following inclusion criteria were enrolled: (1) Age > 18 years, (2) new diagnosis or suspected or confirmed relapse of multiple myeloma following IMVG criteria; and (3) complete WB-MRI acquired along MY-RADS guidelines.

Exclusion criteria were as follows: (1) Age < 18 years; (2) concomitant presence of a malignancy other than myeloma that might cause bone metastatic disease; and (3) incomplete WB-MRI study or study affected by major artifacts.

### 2.2. Image Analysis

Three radiologists with different levels of experience in WB-MRI were selected, including one expert radiologist (AR) with more than 5 years in WB-MRI and 15 years’ experience in MRI, one young radiologist (AC) with 3 years’ experience in MRI and 6 months experience in WB-MRI, and one senior radiology resident (EA) with 6 months experience in general MRI and 3 months experience in WB-MRI in myeloma patients, with MY-RADS teaching and a soft skill course on WB-MRI. Readers with different levels of experience were intentionally selected to reflect real-world variability in clinical practice and to allow the evaluation of the reproducibility of MY-RADS across heterogeneous backgrounds.

The three readers independently completed a clinical reporting template for each WB-MRI according to the MY-RADS reporting system, evaluating images on a Picture Archiving and Communication System (PACS, AGFA HealthCare Enterprise Imaging 8.4, Mortsel, Belgium). Before evaluating WB-MRIs, all radiologists knew the patient’s clinical and laboratory situation, which is essential for interpreting the investigation. Morphological images (fat- and fluid-sensitive sequences, low and high b-value DWIs) and quantitative ADC and rFF% maps are evaluated on PACS workstations by “linking” and browsing them in the different planes using multiplanar registration.

The analysis was conducted following MY-RADS guidelines in each of the seven different anatomical regions (skull, cervical spine, thoracic spine, lumbo-sacral spine, pelvis, thorax, and limbs, including the femurs and the proximal humeri). Presence of para-medullary and extra-medullary disease was also assessed. Possible patterns of disease of bone marrow infiltration included negative, focal, diffuse, focal on diffuse, and micronodular. Diffuse pattern infiltration of bone marrow was evaluated as the most severe pattern observed among the assessed anatomical regions (excluding para-medullary and extra-medullary sites). The patterns were ranked from least to most severe as follows: negative, micronodular, focal, focal on diffuse, and diffuse.

Patients who received a follow-up WB-MRI scan were also evaluated with response assessment categories (RAC) following the indications expressed in MY-RADS [13]. MY-RADS guidelines suggest identifying 2 RACs for each region in order to document heterogeneous response (mixed/discordant responses): a primary RAC is expressed for the pattern seen in most lesions, and a secondary RAC is attributed to the second most represented pattern. Primary and secondary RAC scores from 1 to 5 were expressed for each anatomical segment as before. Overall skeletal response, for both primary and secondary RAC scores, was defined as the worst RAC score observed across all anatomical segments, excluding para-medullary and extra-medullary sites, to reflect the most severe disease behavior at follow-up.

All data were subsequently collected in a single database to assess the inter-reader agreement both for baseline evaluation and for response assessment.

### 2.3. Quantitative Analysis

To further evaluate the reproducibility of ADC and rFF% values among different radiologists, a quantitative analysis was conducted. For patients with follow-up, up to five bone lesions per patient, each with a minimum diameter of 1 cm [13,25,26], were contoured with a 2D circular Region of Interest (ROI) covering the central part of the lesion to assess the consistency of ADC and rFF% values among readers. All measurements were performed on PACS. To ensure that the same lesions were assessed by all readers, the first reader selected lesions from anywhere in the acquisition volume, prioritizing larger lesions and avoiding artifacts (such as nonphysical water diffusion values for ADC), and then tagged them on PACS. The second and third readers then performed their analysis independently on each selected lesion. Data of each ROI, including area (cm^2^), ADC value (µm^2^/s), and fat content (%), were collected from the three readers. All data were subsequently collected in a single database to assess the inter-reader agreement both for ADC and rFF% values.

### 2.4. Study Design and Data Analysis

The observers involved in the study independently evaluated the imaging data and classified the disease patterns. The analysis focused on comparing the agreement between raters beyond what could be expected by chance alone. To quantify this agreement, three main metrics were used:

- Percentage Agreement: Measures the raw percentage of cases where all raters agreed on the classification.
- Cohen’s Kappa: Corrects for agreement occurring by chance, providing a measure of inter-rater reliability.
- Brennan and Prediger’s Coefficient: Another chance-corrected measure of agreement, particularly useful when Kappa may underestimate agreement in cases of high observed agreement.

The agreement between the raters was analyzed across different anatomical regions, and the results were reported with 95% confidence intervals (CI) to indicate the precision of the agreement estimates. The strength of agreement was interpreted using the following categories: Excellent agreement, 0.81–1.00; Substantial agreement, 0.61–0.80; Moderate agreement, 0.41–0.60; Fair agreement, 0.21–0.40; and Slight agreement, 0.00–0.20.

The mean ADC and rFF% values contoured by different readers were assessed with an interclass correlation coefficient (ICC), based on a two-way mixed-effects model, consistency type, with a single measurement per rater. An ICC < 0.5 was considered poor, 0.5–0.75 moderate, 0.75–0.9 good, and >0.9 excellent. For each imaging biomarker (ADC and rFF%), all measurements obtained by the three independent observers were pooled into a single distribution. Descriptive statistics were then calculated across the entire dataset, including mean, standard deviation, and minimum and maximum values.

All statistical analyses were performed with STATA 15, MedCalc (v. 12.1.0 for Microsoft Windows 2000/XP/Vista/7; MedCalc Software, Ostend, Belgium), and IBM SPSS Statistics (v 30.0.0, IBM Corp. Armonk, NY, USA).

## 3. Results

### 3.1. Study Population

Within the PACS of our institution, 70 patients were identified to meet the inclusion criteria 1 and 2; of these, 15 had a WB-MRI negative for bone lesions, and 2 had an incomplete exam, so they were excluded from the final analysis. Of these 53 patients, 1 more was excluded for the presence of major artifacts due to obesity.

**Figure 1:**
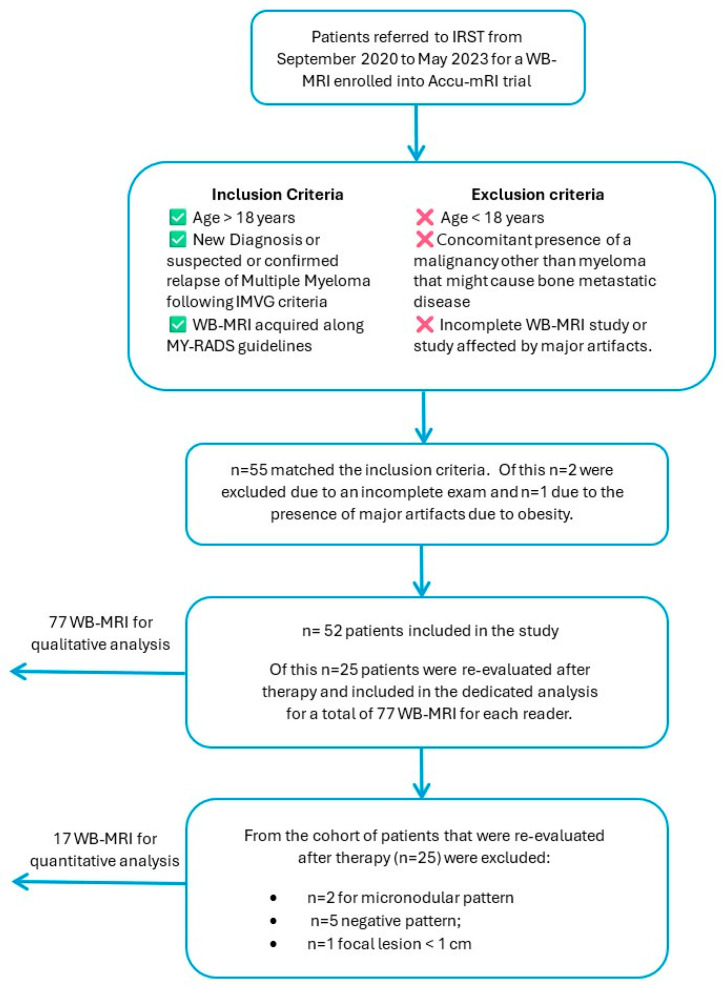
Flowchart of patient’s selection criteria.

Eventually, 52 patients [23 Female, 29 Male; Mean age: 61 years (range 40–81 years); Isotype: IgA (12), IgG (32), Micromolecular (2), Non secretory (6)] were included in the study because of diagnosed with active multiple myeloma due to IMWG criteria (MM, 47) or presence of clinic lab data compatible with relapse-refractory MM (RR, 5). In total, 25 (20 MM, 5 RR) patients were evaluated after therapy and included in the dedicated analysis. A total of 77 WB-MRIs were evaluated by each reader. The 25 patients with paired WB-MRIs were further investigated for quantitative analysis MRI (qMRI) studies, but following the recommendations of MY-RADS, in order to improve stability and reliability of the measurements, patients with lesions < 1 cm were excluded: of these, eight patients were excluded for the presence of a micronodular pattern (two), negative pattern (five), amd focal lesion < 1 cm (one). In total, 17 patients with a focal or diffuse pattern of bone marrow infiltration met the criteria for inclusion in the qMRI study and were finally evaluated. Across these 17 patients, 143 final lesions were quantified both in ADC and in rFF% maps (Figure 1).

### 3.2. The Analysis of Inter-Observer Agreement at Staging

**Figure 2:**
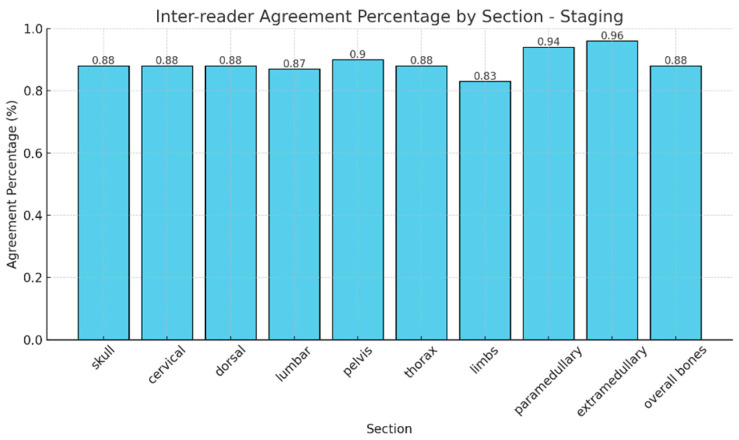
Agreement percentages in the identification of different patterns of bone marrow infiltration across anatomical regions.

The observed agreement in the identification of different patterns of bone marrow infiltration across anatomical regions was consistently high among readers, ranging from 83% to 96% (Figure 2). The overall skeletal agreement was 88%. Cohen’s Kappa shows moderate to substantial inter-reader agreement across most regions, with values ranging from 0.66 to 0.86. The lowest value was observed in the extramedullary region (0.23, 95% CI −0.08–0.54), reflecting low concordance in this area. This result is likely influenced by the small number of positive cases (n = 2) and a single misclassification by a less experienced reader, as well as the known sensitivity of Kappa to skewed marginal distributions.

Brennan and Prediger values were higher than Kappa values, ranging from 0.79 to 0.92, and indicating substantial agreement across most regions (Table 1). These values tend to be more stable in the presence of class imbalance and confirm the overall trend of high inter-reader reproducibility. The results of ADC and rFF% values in each anatomical region (cervical, dorsal, lumbar, pelvis, thorax, and limbs), as well as for the whole skeleton, are summarized in Supplementary Table S1, which reports the mean, standard deviation, minimum, maximum, and range for both ADC and rFF%.

### 3.3. The Analysis of Inter-Observer Agreement at Follow-Up

**Figure 3:**
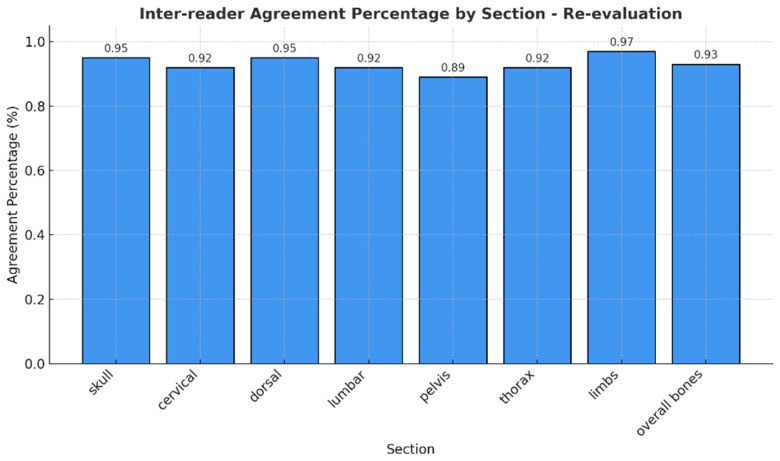
Agreement percentages in the identification of RAC across anatomical regions.

Notably, no mixed responses were observed by any of the readers across all regions, so we considered only one RAC for each anatomic segment. The observed agreement of different RAC across the evaluated anatomical regions was consistently high, with percentages ranging from 89% (pelvis) to 97% (arms) (Figure 3). Cohen’s Kappa values indicated substantial to excellent agreement, ranging from 0.83 in the pelvis to 0.95 in the arms. Similarly, Brennan and Prediger’s agreement coefficients supported these findings, with values ranging from 0.87 (pelvis) to 0.97 (arms) (Table 2). The confidence intervals for both Kappa and Brennan and Prediger’s coefficients were narrow, reflecting high precision and further supporting the reliability of the assessments, particularly in the cranium, dorsal, and overall bone evaluations.

**Figure 4:**
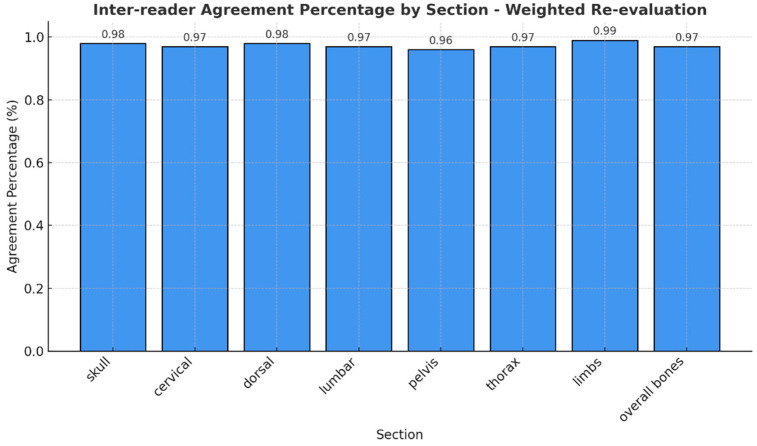
Different agreement percentages for the class of RAC.

If we consider that RAC 1 and 2 indicate response, RAC 3 indicates stable disease, and RAC 4 and 5 indicate disease progression, it is reasonable to consider that disagreement between adjacent categories (RAC 1 and 2 versus RAC 3 versus RAC 4 and 5) is less severe than disagreement between distant categories. To account for this, we evaluated a weighted index that considers the relative severity of disagreement across RACs (Figure 4).

Percentage agreement for the weighted data is consistently high across all regions, ranging from 94% (pelvis and some areas in the thorax) to 99% (arms). Cohen’s weighted kappa indicates strong agreement, with values ranging from 0.88 (pelvis) to 0.96 (arms), and Brennan and Prediger’s weighted agreement coefficients are similarly high, confirming strong inter-observer agreement across all regions.

### 3.4. Quantitative Analysis

We evaluated a total of 143 lesions, respectively, localized in: cervical spine (13), dorsal spine (20), lumbar spine (42), pelvis (28), limbs (17), thorax (21), and skull (2). The only two lesions of the skull were excluded from the analysis due to the statistical insignificance. The final analysis considers 141 lesions.

**Figure 5:**
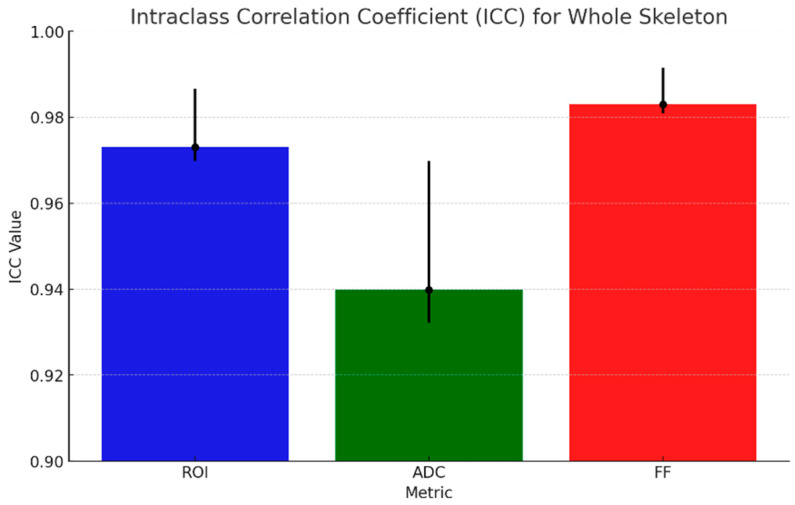
ICC (black dots) for ADC, rFF%, and ROI area with 95% CI (black lines).

**Figure 6:**
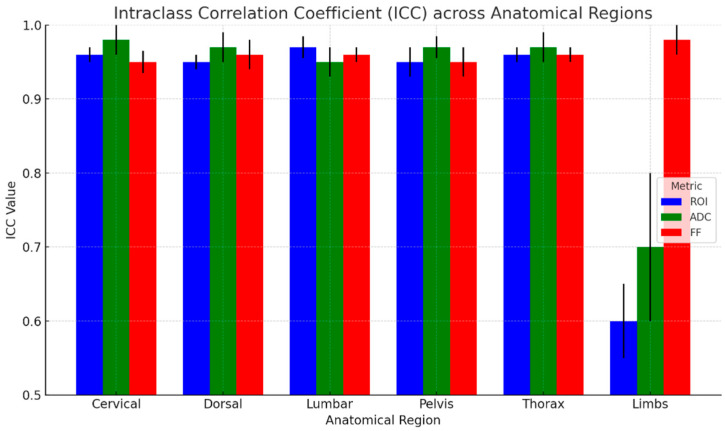
ICC across anatomical regions with 95% CI (black lines).

The results of ICC analysis on ADC and rFF% values and ROI area are graphically shown in Figure 5 for all the anatomical regions taken into consideration, and the detailed regions are shown in Figure 6. We report excellent agreement among the three radiologists in both ROI area, ADC, and rFF% values, except for the limbs, where we obtained a moderate agreement of 0.63 for ROI area, a good agreement for ADC values, and an excellent agreement for rFF% values.

Considering the whole skeleton, the ICC values for whole-skeleton measurements indicate excellent inter-observer reliability across all metrics. The highest agreement was observed for rFF% with an ICC of 0.98 (95% CI: 0.98–0.99), and the ADC showed slightly lower but still strong agreement, with an ICC of 0.94 (95% CI: 0.93–0.97).

## 4. Discussion

Our results indicate substantial to excellent agreement across all metrics, confirming the reproducibility of MY-RADS assessments among radiologists with different experience levels. Importantly, high agreement was observed for focal lesions, which is critical for early detection and management of bone marrow infiltration in MM as well as for application of the SLiM CRAB criteria [27].

The inter-reader agreement observed in our study is consistent with findings by Croft et al. [22], who evaluated interobserver agreement between three expert radiologists (>8 years of experience) in baseline WB-MRI in myeloma. They reported an overall skeleton ICC of 0.91, demonstrating excellent agreement in the assessment of myeloma-related bone disease, but their ICC varied between skeletal regions, with spine, pelvis, and ribs showing good inter-observer agreement, whereas skull and long bones were moderate. In contrast to their study, we did not observe variations in agreement across different anatomical regions, even if we observed that the senior resident missed some lesions, particularly in the paramedullary and extramedullary areas, highlighting the complexity of evaluating soft tissue involvement in MM and enhanced radiologist training. This supports the inclusion of readers with different expertise levels, reflecting routine clinical practice where WB-MRI may be interpreted by radiologists with heterogeneous backgrounds. Our findings indicate that MY-RADS criteria ensure good reproducibility in this setting, although experience remains particularly relevant in complex regions.

Furthermore, our results align with those of Pricolo et al. [28], who studied inter-observer agreement using the MET-RADS-P system in metastatic prostate cancer between three radiologists of different levels of expertise. Their findings of excellent agreement in bone assessments between radiologists support a high reproducibility of MY-RADS and MET-RADS-P criteria. However, they found lower agreement, particularly in node evaluation and in the assessment of secondary RAC, underlining the importance of experience and continuous education in improving the accuracy of WB-MRI assessments, particularly in these areas.

Lai et al. [29] highlighted the superior consistency of WB-MRI over Whole-Body Low-Dose CT for experienced observers; as a matter of fact, interobserver agreement for WB-MRI was superior to WB-CT overall and for each region, for inexperienced observers (ICC 0.98 versus 0.77, 95%CI 0.96–0.99 versus 0.45–0.91) and for inexperienced observers (ICC 0.95, 95%CI 0.72–0.98) than on WB-CT (ICC 0.72, 95%CI 0.34–0.88), but with a less clear benefit.

This study confirms the need for expertise and that the overall reliability of WB-MRI in assessing myeloma burden is high.

Moreover, Lecouvet et al. [30] demonstrated that MRI has a better reproducibility rather than PET-CT; an intra- and inter-reader agreement very good for MRI (64 axial MRI and 20 WB-MRI) (k = 0.90 [0.81; 1.00] and 0.88 [0.78; 0.98]), whilst intra- and inter-reader agreement was good for PET/CT (k = 0.80 [0.69; 0.91] and 0.71 [0.56; 0.86]). MRIs were evaluated by two radiologists with respectively > 25 and >3 years of expertise.

In all articles, the expertise of the radiologists played a significant role. Experienced readers showed higher consistency compared to junior readers, particularly in complex regions. This suggests that the variability in regions like the limbs or skull and soft tissue could be improved with more experience, as suggested by Berardo and colleagues, who demonstrated that 80 WB-MRI reported in myeloma leads to a high level of inter-observer concordance [31].

Our study’s second section examines readers’ agreement while utilizing 2D ROIs to assess ADC and rFF% values, which are becoming more and more recognized as potential imaging biomarkers.

At present, there is clear data supporting the use of rFF% for evaluation of liver steatosis that could represent a marker of metabolic-associated fatty liver disease (MAFLD), and a consensus profile has recently been published [32]. Furthermore, Schmeel et al. [33] demonstrated the extreme accuracy, consistency, and reproducibility of MRI-based measurement of vertebral bone marrow using rFF% across readers, field strengths, and MRI platforms, demonstrating its use as a quantitative imaging biomarker in multicentric research.

Moreover, ADC is emerging as a possible biomarker for clinical practice, and the Diffusion-Weighted Imaging Biomarker Committee of the Quantitative Imaging Biomarkers Alliance (QIBA) has recently published a report that shows that the increase in ADC values beyond a certain percentage, differing from organ to organ, indicates true changes with 95% confidence [27]. Although bone lesion evaluation is not included in these QIBA guidelines, their quantitative assessment in accordance with the MY-RADS could be useful, and recently published studies about the use of ADC and rFF% evaluation of bone lesions enhance this application [16,18,34,35,36].

We observed an excellent interobserver agreement both in the evaluation of ADC values and in rFF% across all anatomic regions. Our data are substantially in agreement with some articles published about the reproducibility of these values.

Castagnoli et al. [18] found excellent inter-reader agreement (ICC = 0.95) reported by on rFF% evaluation of normal marrow (89.76%) and malignant bone lesions from breast (14.46%), myeloma (13.12%), and prostate cancer (13.67%) (*p* > 0.017). The excellent inter-reader agreement reported aligns with our findings, supporting the reliability of rFF% measurements across different anatomical regions. Similar results on high reproducibility were found on ADC values by Michoux et al. [17], which highlights the repeatability and reproducibility limits for ADC measurements in an oncologic multicentre setting using WB-MRI. According to their work, for an ADC change to be considered clinically relevant, it must exceed specific thresholds, which vary depending on the organ, but the coefficient of variation in ADC was not influenced by other factors (center, reader). Small variations in ADC measurements, especially in regions such as bone marrow and complex tissues, may not necessarily reflect real pathological changes but could be attributed to intrinsic reproducibility limits.

Moreover, Giles et al. [23] demonstrated that WB-MRI is a sensitive and repeatable tool for monitoring treatment response in myeloma, with ADC values showing low variability and clear differentiation between responders and non-responders.

Similarly, a test–retest study [35] in advanced prostate cancer confirmed the repeatability of mean ADC and rFF% values in both individual lesions and overall disease burden, reinforcing their potential as reliable quantitative biomarkers in metastatic bone disease.

The good results of reproducibility of ADC and rFF% found in our study could support the 3T scanner’s ability to perform multiparametric WB-MRI as suggested in the OPTIMUM/MUKnine trial, a multicentric trial performed in England to improve WB-MRI in myeloma patients in different scanners [37,38].

Evaluating inter-reader ROI placement was crucial to our study, as ROI shape and positioning significantly impact ADC reproducibility—an insight supported by [39] et al., who found narrow band-shaped ROIs offered the best repeatability. Although we focused on oncologic lesions, whereas Møller et al. investigated inflammatory lesions in the sacroiliac joints and used circular ROIs, our findings similarly highlight the importance of anatomical adaptation in ROI design, especially given reduced agreement in limb lesions. Our use of standardized, centrally placed 2D circular ROIs aligns with MY-RADS and the recent QIBA Profile [25], which emphasizes consistent, well-defined ROI strategies to minimize variability. By pre-selecting lesions and guiding readers via PACS markers, we improved consistency and met QIBA reproducibility benchmarks (coefficients of variation < 10%). Nonetheless, lower agreement in complex regions like limbs suggests future value in adaptive or semi-automated ROI strategies. Overall, our approach supports QIBA-aligned protocols to enhance the reliability of ADC as a quantitative imaging biomarker in multi-center oncologic studies.

This study has several limitations. First, the sample size was relatively small, particularly in the follow-up subgroup, which may limit the statistical power and generalizability of the findings. The low number of patients undergoing follow-up WB-MRI may have limited the range of clinically relevant scenarios captured in our cohort and could also explain the wide confidence intervals observed in some analyses. This underlines the need for larger, multicenter studies to confirm and generalize our results. Second, the majority of detectable lesions were osseous, with limited representation of soft tissue involvement—only two patients presented with extramedullary disease and nine with paramedullary infiltration. This may reduce the applicability of our findings to patients with a broader spectrum of disease presentations. Finally, no cases of mixed response or secondary response assessment categories (RACs) differing from the primary RAC were observed, which limits the evaluation of inter-reader agreement in these more complex and clinically challenging scenarios.

## 5. Conclusions

Our research shows that whole-body MRI, using the MY-RADS criteria, offers a solid framework for consistent evaluation of multiple myeloma. The reproducibility of this method supports its incorporation into clinical practice for patient staging and management. Additionally, the reproducibility of quantitative ADC and rFF% measurements in our study supports their use as reliable biomarkers for disease assessment. These results emphasize the importance of protocol standardization and radiologist training to enhance multiparametric WB-MRI diagnostic consistency. This can be achieved through structured training programs focused on MY-RADS criteria and “functional” imaging sequences like ADC and rFF%.

## Figures and Tables

**Table 1:** Different statistical evaluations of agreement in different anatomical regions.

Region	% Agreement	Kappa	Kappa 95% CI	Brennan and Prediger	B&P 95% CI
Cranium	88%	0.66	0.49–0.82	0.85	0.75–0.94
Cervical	88%	0.82	0.71–0.93	0.86	0.77–0.94
Dorsal	88%	0.84	0.74–0.94	0.86	0.77–0.94
Lumbar	87%	0.81	0.70–0.92	0.83	0.73–0.93
Pelvis	90%	0.86	0.77–0.95	0.87	0.79–0.96
Thorax	88%	0.83	0.73–0.93	0.86	0.77–0.94
Limbs	83%	0.73	0.61–0.85	0.79	0.69–0.89
Overall Skeleton	88%	0.84	0.74–0.93	0.86	0.77–0.94
Paramedullary	94%	0.74	0.52–0.96	0.87	0.76–0.98
Extramedullary	96%	0.23	−0.08–0.54	0.92	0.84–1

**Table 2:** Different statistical evaluations of agreement in different anatomical regions between RAC.

Region	% Agreement	Kappa	Kappa 95% CI	Brennan and Prediger	B&P 95% CI
Cranium	95%	0.91	0.79–1	0.96	0.84–1
Cervical	92%	0.87	0.74–1	0.90	0.79–1
Dorsal	95%	0.92	0.80–1	0.93	0.84–1
Lumbar	92%	0.88	0.74–1	0.90	0.79–1
Pelvis	89%	0.83	0.67–0.99	0.87	0.74–1
Thorax	92%	0.87	0.72–1	0.90	0.79–1
Limbs	97%	0.95	0.86–1	0.97	0.90–1
Overall Skeleton	92%	0.88	0.75–1	0.90	0.79–1

## Data Availability

Data is contained within the article or Supplementary Material.

## Supplementary Materials

The following supporting information can be downloaded at: https://www.mdpi.com/article/10.3390/diagnostics15212715/s1, Table S1: descriptive statistics of quantitative analysis; Table S2: raw data.

## Abbreviations

The following abbreviations are used in this manuscript:

WB-MRI	WholeWhole-Body Magnetic Resonance Imaging
MY-RADS	Myeloma Response Assessment and Diagnosis System
MM	Multiple Myeloma
WB-LD-CT	Whole-Body Low-Dose Computed Tomography
PET-CT	Positron Emission Tomography-Computed Tomography
FDG	18F-fluorodeoxyglucose
DWI	Diffusion Weighted Images
ADC	Apparent Diffusion Coefficient
rFF%	Relative Fat Fraction
RAC	Response Assessment Categories
IMVG	International Myeloma Working Group
